# *Ferula latisecta* gels for synthesis of zinc/silver binary nanoparticles: antibacterial effects against gram-negative and gram-positive bacteria and physicochemical characteristics

**DOI:** 10.1186/s12896-024-00878-x

**Published:** 2024-08-01

**Authors:** Ali Es-haghi, Mohammad Sadegh Amiri, Mohammad Ehsan Taghavizadeh Yazdi

**Affiliations:** 1 Department of Biology, Mashhad Branch, Islamic Azad University, Mashhad, Iran; 2 Department of Biology, Payame Noor University, Tehran, Iran; 3 Applied Biomedical Research Center, Mashhad University of Medical Sciences, Mashhad, Iran

**Keywords:** Zinc oxide nanoparticles, ZnO NPs, Silver, *Ferula latisecta*, Plant gels, Biocompatibility

## Abstract

This study explores the potential antibacterial applications of zinc oxide nanoparticles (ZnO NPs) enhanced with silver (Ag) using plant gel (ZnO-AgO NPs). The problem addressed is the increasing prevalence of pathogenic bacteria and the need for new, effective antimicrobial agents. ZnO NPs possess distinctive physicochemical properties that enable them to selectively target bacterial cells. Their small size and high surface area-to-volume ratio allow efficient cellular uptake and interaction with bacterial cells. In this study, the average size of the synthesized ZnO-Ag nanoparticles was 77.1 nm, with a significant standard deviation of 33.7 nm, indicating a wide size distribution. The nanoparticles demonstrated remarkable antibacterial efficacy against gram-negative and gram-positive bacteria, with inhibition zones of 14.33 mm for *E. coli* and 15.66 mm for *B. subtilis* at a concentration of 300 µg/ml. Minimum inhibitory concentrations (MIC) were determined to be 100 µg/ml for *E. coli* and 75 µg/ml for *S. saprophyticus*. Additionally, ZnO-Ag NPs exhibited excellent biocompatibility, making them appropriate for various pharmacological uses. This study utilizes *Ferula latisecta* gels, offering a sustainable and eco-friendly approach to nanoparticle synthesis. Incorporating of Ag into ZnO NPs significantly enhances their antimicrobial properties, with the combined results showing great inhibition effects on pathogenic microbes. The findings suggest that ZnO-Ag NPs could be a promising candidate for addressing the challenges posed by drug-resistant bacterial infections and enhancing antimicrobial treatments.

## Introduction

Various types of commercial metal oxide nanoparticles have been utilized in biomedical applications [1]. Fe₃O₄ (Magnetite) has been employed for drug delivery, MRI contrast agents, and hyperthermia treatment due to its high magnetic properties and biocompatibility, although potential toxicity and stability issues have been noted. CeO₂ (Cerium Oxide) has been used for antioxidant therapy, neuroprotection, and anti-inflammatory purposes, praised for its excellent antioxidant properties and biocompatibility, but concerns about long-term toxicity and organ accumulation have been raised. Al₂O₃ (Alumina) has found applications in dental implants, bone tissue engineering, and wound healing, valued for its high mechanical strength and biocompatibility, yet potential cytotoxicity and limited biodegradability have been reported. SiO₂ (Silica) has been applied in drug delivery, biosensing, and imaging, appreciated for its high surface area, biocompatibility, and ease of functionalization, however potential toxicity at high doses and stability issues have been observed. MnO₂ (Manganese Dioxide) has been utilized as MRI contrast agents, in drug delivery, and cancer therapy, recognized for its high MRI contrast and catalytic activity, but toxicity and stability issues have been identified. WO₃ (Tungsten Oxide) has been used in photothermal therapy and biosensing, noted for its high photothermal conversion efficiency and biocompatibility, but limited research on long-term effects and potential toxicity have been mentioned. Additionally, composites such as Fe₃O₄/SiO₂ (Magnetite/Silica) and ZnO/Ag (Zinc Oxide/Silver) have been employed, combining properties of individual components to enhance biomedical applications, however complex synthesis and potential toxicity should be investigated (Table 1).

**Table 1:** Various type of metal oxide NPs in biomedicine

Metal Oxide NP	Biomedical Applications	Strengths	Limitations	Ref
Fe₃O₄ (Magnetite)	Drug delivery, MRI contrast agents, hyperthermia treatment	High magnetic properties, biocompatibility	Potential toxicity, stability issues	[2]
CeO₂ (Cerium Oxide)	Antioxidant therapy, neuroprotection, anti-inflammatory	Excellent antioxidant properties, biocompatibility	Long-term toxicity, accumulation in organs	[3]
Al₂O₃ (Alumina)	Dental implants, bone tissue engineering, wound healing	High mechanical strength, biocompatibility	Potential cytotoxicity, limited biodegradability	[4]
SiO₂ (Silica)	Drug delivery, biosensing, imaging	High surface area, biocompatibility, easy functionalization	Potential toxicity at high doses, stability issues	[5]
MnO₂ (Manganese Dioxide)	MRI contrast agents, drug delivery, cancer therapy	High MRI contrast, catalytic activity	Potential toxicity, stability issues	[6]
WO₃ (Tungsten Oxide)	Photothermal therapy, biosensing	High photothermal conversion efficiency, biocompatibility	Limited research on long-term effects, potential toxicity	[7]
ZrO₂ (Zirconium Dioxide)	Dental implants, bone tissue engineering, biosensing	High mechanical strength, biocompatibility	Potential cytotoxicity, limited biodegradability	[8]
SnO₂ (Tin Oxide)	Biosensing, gas sensing, cancer therapy	High sensitivity, biocompatibility	Potential toxicity, stability issues	[9]
MgO (Magnesium Oxide)	Antibacterial, wound healing, cancer therapy	High antibacterial activity, biocompatibility	Potential cytotoxicity, limited research on long-term effects	[10]
NiO (Nickel Oxide)	Cancer therapy, biosensing	High catalytic activity, biocompatibility	Potential toxicity, stability issues	[11]
CuO (Copper Oxide)	Antimicrobial, anticancer, antioxidant, wound healing	Strong bactericidal properties, low cost	Potential toxicity, stability issues	[12]
TiO₂ (Titanium Dioxide)	Photodynamic therapy, drug delivery, biosensing, cancer therapy	High photocatalytic activity, biocompatibility	Potential toxicity, stability issues	[13]
Fe₃O₄/SiO₂ (Magnetite/Silic nanoComposite)	Drug delivery, MRI contrast agents, hyperthermia treatment	Combined magnetic and biocompatibility properties	Complex synthesis, potential toxicity	[14]
ZnO/Ag (Zinc Oxide/Silver NanoComposite)	Antimicrobial, wound healing, cancer therapy	Enhanced antibacterial activity, biocompatibility	Potential toxicity, stability issues	[15]
ZnO/CuO Nanocomposite	Antibacterial, wound healing	Enhanced antibacterial activity, Cytocompatibility	Potential toxicity, stability issues	[16]
ZnO/CuO Nanoparticles	Wound dressing, bactericidal	Enhanced antibacterial activity, Cytocompatibility	Potential toxicity, stability issues	[17]
ZnO–CuO nanocomposites encapsulated calcium and carbon	Antimicrobial properties	Enhanced antibacterial activity against non-MDR and MDR skin pathogens.	Molecular studies	[18]
ZnO/C/Ca nanocomposites	Antimicrobial properties	Enhanced antibacterial activity against skin ulcer pathogens	Potential toxicity, stability issues	[19]
Copper oxide nanoparticles	Wound dressing activity	Cytocompatibility with strong bactericidal properties for wound dressing material	Potential toxicity, stability issues	[20]

Among them, Zinc Oxide Nanoparticles (ZnO NPs) have exhibited remarkable promise in environmental and biomedical treatment, primarily attributable to their exceptional attributes [21–24]. These nanoparticles inherently possess potent antimicrobial characteristics and have the unique capability to discriminate in their assault, sparing healthy cells while targeting bacterial cells [25–27]. ZnO NPs have demonstrated the capacity to programmed cell death in bacteria cells, impede their uncontrolled proliferation, and disrupt the growth of bacterial cells [28–30]. Moreover, their impact on normal cells is exceptionally mild, rendering them an exceptionally favorable candidate for advancing cancer therapy [31–33]. The integration of silver (Ag) into ZnO NPs has revealed a marked enhancement in their antimicrobial prowess [34, 35]. Silver ions have been recognized for their antimicrobial and anticancer attributes [36–38]. When combined with ZnO NPs, the amalgamation of silver ions and ZnO NPs results in a synergistic augmentation of cytotoxicity against bacteria cells. The presence of silver ions elevates the production of reactive oxygen species (ROS) within bacteria cells, ultimately inducing escalated oxidative stress and, consequently, cell mortality [39–41]. This combination elevates the effectiveness of ZnO NPs in the context of antimicrobial treatments significantly.

Plant extracts play a pivotal role in the intricate synthesis process of NPs, and they exert a notable influence on the antimicrobial properties of the ultimate nanomaterial [42–44]. Within plant materials reside a bounty of bioactive compounds, including polyphenols, flavonoids, and terpenoids, each replete with antioxidant and anticancer attributes [45–47]. These bioactive compounds take on the roles of stabilizing agent throughout the synthesis of ZnO NPs, exerting their influence on the size, morphology, and surface characteristics of these nanoparticles. The incorporation of plant extracts into the synthesis process serves to amplify the anticancer efficacy of ZnO NPs by imparting additional bioactive compounds to the nanomaterial [48–51].

The genus *Ferula* (Apiaceae) comprises 221 accepted species and members of this genus are distributed from the Canary Islands in the west through the Mediterranean region, Middle East and Central Asia to western China in the east and northern India in the south [52, 53]. *Ferula latisecta* Rech.f. & Aellen, is one of the species of this genus with a characteristic strong odor which commonly known as “Koma Hezar-Masjed” and “Sasekoma” in Iran. It is an endangered and medicinal species growing on clay and marl hills and is distributed in a small area of Iran and Turkmenistan [52, 54, 55]. The oleo-gum-resin of *F. latisecta* were collected from Zarrin-Kuh Protected Area, Razavi Khorassan Province (NE Iran). A voucher specimen was identified and deposited (No. 477) in Dargaz Payame Noor University Herbarium. *Ferula latisecta* has shown a broad spectrum of ethnomedicinal applications and has been widely used in Iranian Traditional Medicine. In the folk medicine of northeast Iran, *F. latisecta* is used for treating parasitic diseases, relieve infant stomachache, and controlling diabetes. The leaves and young stems are taken for food. Furthermore, it is applied for the treatment of digestive system diseases, and as anthelmintic [56, 57].

The process of integrating silver into ZnO NPs through the use of plant extracts is an intricate two-step endeavor. Initially, ZnO NPs are painstakingly synthesized, employing a plant extract as a stabilizing agent [58, 59]. This typically needs the precise mixing of a zinc precursor with the plant extract under controlled conditions, followed by the meticulous reduction of the precursor, yielding ZnO NPs. In the subsequent step, silver ions are introduced into the solution housing the ZnO NPs using a silver precursor. The silver ions engage with the ZnO NPs, culminating in the ZnO-Ag nanocomposites. The plant extract assumes a pivotal role in both the reduction of the zinc precursor and the stabilization of the ensuing nanocomposites [60, 61].

The union of ZnO NPs and silver demonstrates the potential to surmount the challenges posed by drug resistance in bacteria cells [62]. The development of drug resistance is a formidable obstacle in the microbes’ treatment, as bacteria cells progressively build resistance to drugs [63, 64]. The combination of ZnO NPs and silver offers an innovative strategy to confront drug resistance. The synergistic effects generated by ZnO NPs and silver ions trigger cell demise through several mechanisms, rendering it arduous for bacteria cells to develop resistance [65–67].

Therefore, Zinc Oxide Nanoparticles (ZnO NPs) present considerable potential within the ambit of antimicrobial treatment, owing to their selective targeting and inherent microbial attributes. Within this exploration, integrating of silver (Ag) into ZnO NPs amplifies their antibacterial efficacy via synergistic interplay. Plant extracts wield a profound influence during the synthesis process, shaping the characteristics of the ultimate nanomaterial. The union of ZnO NPs and silver hints at the promise of overcoming drug resistance mechanisms, charting a promising path for future antimicrobial therapies.

This study’s innovative aspects were underscored by several key points. An eco-friendly synthesis method was employed, utilizing *F. latisecta* gels as a sustainable and eco-friendly stabilizing agent for synthesis of zinc oxide nanoparticles (ZnO NPs) enhanced with silver (Ag). This method circumvented chemical regeneration agents typically required in nanoparticle synthesis, adhering to green chemistry principles. The antimicrobial properties of the nanoparticles were significantly enhanced by the incorporation of silver. Silver ions synergized with ZnO NPs to augment cytotoxicity against bacterial cells through an increased production of reactive oxygen species (ROS), which induced oxidative stress and cell death. Furthermore, plant extracts were used as stabilizing agents, introducing bioactive compounds such as polyphenols, flavonoids, and terpenoids into the nanoparticles. These compounds not only stabilized the nanoparticles but also amplified their anticancer and antimicrobial properties, adding therapeutic dimension. A dual-phase synthesis process was implemented, wherein ZnO NPs were first synthesized using plant extracts, followed by the incorporation of silver ions. This method ensured the effective integration of silver into the ZnO nanoparticles, culminating in a composite material with superior properties. Lastly, combining of ZnO and Ag in the nanoparticles presented a novel approach to addressing bacterial drug resistance. The synergistic effects of ZnO NPs and silver ions obstructed the development of resistance by bacteria, offering a promising strategy for future antimicrobial therapies.

## Experimental section

### Synthesis and characterization of nanoparticles

**Fig. 1:**
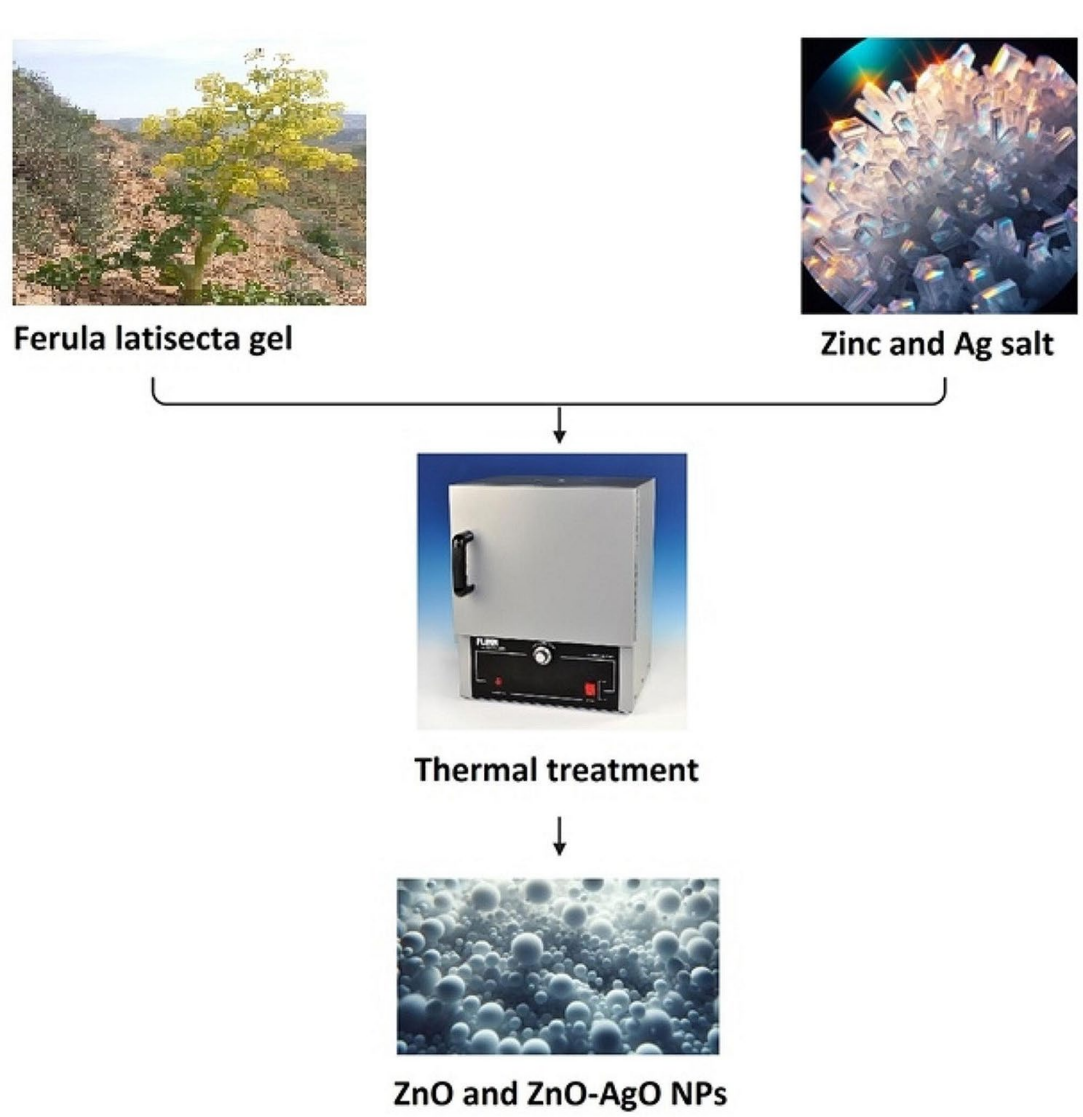
Schematic diagram of green synthesis of NPs

Zinc oxide nanoparticles were synthesized using a water extract from *F. latisecta* gels as a capping agent (Fig. 1). Initially, a zinc salt solution was prepared by dissolving zinc nitrate hexahydrate in distilled water to form a 0.1 M solution. *F. latisecta* gels was slowly added to the zinc salt solution, and the mixture was allowed to react at room temperature for 24 h. Subsequently, the reaction mixture was subjected to a thermal treatment at 400 °C for 5 h, resulting in the formation of capped ZnO nanoparticles. These nanoparticles were washed with ethanol and distilled water and then dried in an oven at 60 °C for 12 h. Finally, the nanoparticles were characterized using X-ray diffraction (XRD, Explorer 40 KV), field scanning electron microscopy (FESEM, MIRA3 TESCAN), and Fourier-transform infrared (FTIR, AVATAR 370). In the second synthesis process, nanoparticles composed of zinc oxide and silver were produced. A metal salt solution was prepared by adding 1 mol percent of silver nitrate to the zinc salt solution. After combining the plant gels with the metal salt solution and allowing the reaction to proceed for 24 h at room temperature, the mixture underwent thermal treatment at 400 °C for 5 h. This process led to the synthesis of ZnO-AgO nanoparticles. Following the synthesis, the nanoparticles were washed, dried, and characterized in the same manner as the first process.

### Disc diffusion assay

Gram-positive [*Bacillus subtilis* (PTCC 1365) and *Staphylococcus saprophyticus* ATCC 49,453] and Gram-negative [Escherichia coli ATCC 25,922 and Pseudomonas aeruginosa ATCC 9027] bacteria were employed in this study. Blank discs were impregnated with antibiotics and 300 µg/ml nanoparticles. A bacterial suspension was prepared and used for uniformly seeding bacteria on Mueller–Hinton agar medium [68]. The plates were kept at 37 °C for 24 h. Afterheat, the diameter of the inhibition zone was dignified. Gentamicin was employed as positive control. All experiments were carried out in triplicate.

### Determining MIC and MBC

In order to specify MIC/MBC, the microbroth dilution method was used employing chloramphenicol as a control. Briefly, in a 24-well microplate, bacteria were inoculated with ZnO/Ag nanoparticles at the doses of 0, 25, 50, 75, 100, 125, and 300 µg/mL. Afterheat at 37 °C, the first well in which no growth was detected was reflected as the MIC. To specify MBC, nutrient agar containing plates were inoculated with bacteria from wells in which no growth was observed; the plates were retained at 37 °C (24 h). Colony appearance confirmed the feasibility of the bacteria, and the absence of colony appearance indicated that the bacteria were non-alive at the specific dose (i.e., MBC).

### Investigating the biofilm formation ability

The purpose of this experiment was to evaluate the capability of the bacteria to form a strong biofilm using an altered microtiter plate technique [69]. The alterations introduced were the employ of deionized water for washing in preference to PBS and considering an extended incubation time. For this purpose, of TSB medium (180 µL) was added to wholly of a microplate (96-well), and at that time, deionized water (10µL) was added. As a final point, pure bacterial suspension (10µL) was added to each well, so the volume in each well reached 200 µL. The microplate was retained at 37 °C for 48 h. Then 200 µL of 95% ethanol was added to stabilize the biofilm (15 minutes’ incubation), followed by the adding of 200 µL of 0.025% safranin (10 minutes’ incubation) for staining. After emptying the content of the wells, the microplate was washed three times and lastly allowed to be dried. Next, 200 µL of acetic acid (33%) was added and after 15 min of incubation, the absorbance of each well was document at 492 nm. To ascertain the capability of the bacterial strain to form biofilm, the ODc was determined, representing the ratio of the mean OD plus 3 times the SD of the negative control. The dignified OD was reflected to be the capability of the strain to form biofilm. The experiment was repeated three times for each bacterial strain.

### Anti-biofilm effects of ZnO/Ag nanoparticles

The anti-biofilm effects of ZnO/Ag nanoparticles were examined using the modified microtiter plate method. For this purpose, TSB medium(180µL) was added to all wells, and then serially diluted nanoparticle solutions (10 µL) were added to each well. Lastly, pure bacterial suspension (10 µL) was inoculated into each well. The final volume in each well was 200 µL, and the concentrations of NPs in the wells were according to Table 2. The microplate was retained at 37 °C for 24 h. The tests were repeated three times, and the means of absorbance evaluations were compared.

### Investigating the effects of ZnO/Ag nanoparticles on formed biofilm

To investigate the effects of ZnO/Ag nanoparticles on pre-formed biofilm, 180 µL of TSB medium and 10 µL of the bacterial suspension (with a concentration of 0.5 McFarland) were added to all wells of a 96-well microplate. The microplate was placed in an incubator at 37 °C. After 24 h the microplate was removed from the incubator, and 10 µL of serially diluted ZnO/Ag nanoparticles were added to each well to obtain the final concentrations of 0, 25, 50, 75, 100, 125, and 300 µg/mL. After 3 h of incubation, the content of the microplate was gradually removed. After fixing the bacteria in each well with ethanol and staining with safranin as mentioned previously, the absorbance was document at 492 nm. The biofilm inhibition ratio was estimated using Eq. 1.

1 $${\rm{Biofilm}}\;{\rm{inhibition}}\left( \% \right) = 100 - \left[ {{{{\rm{OD492}}\;{\rm{positive}}\;{\rm{control}}} \over {{\rm{OD492}}\;{\rm{biocide}}}}} \right] \times 100$$

This experiment was repeated 3-times for each bacterium, and average values were considered for comparisons.

### Statistical analysis

Statistical analysis was done in SPSS software version 21. Mean ± SD was compared by one-way ANOVA, and p-values of less than 0.05 were deliberated statistically significant.

## Results and discussion

### Powder X-ray diffraction

**Fig. 2:**
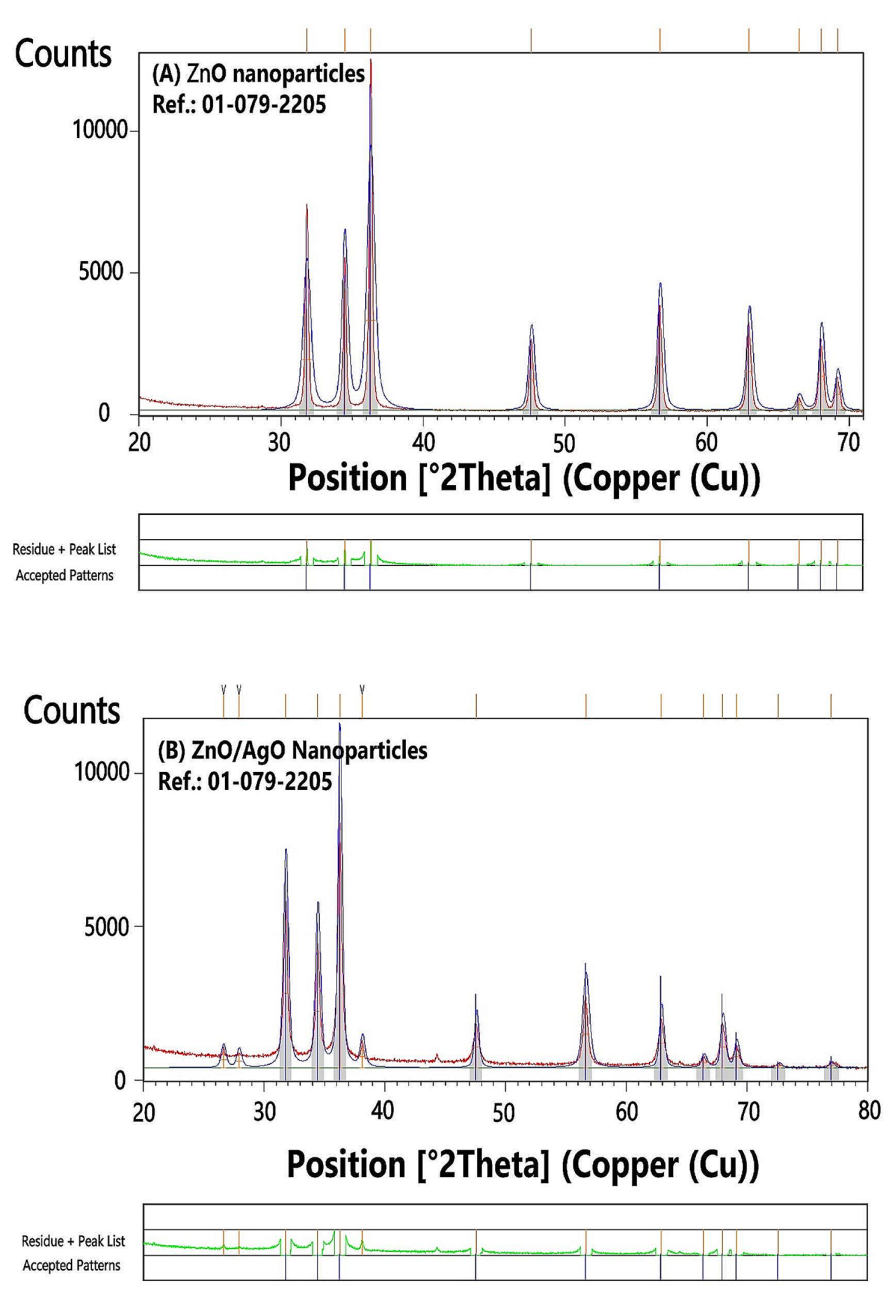
PXRD pattern of ZnO and ZnO-AgO NPs

The remarkable alignment observed between the experimental data derived from PXRD analysis and the calculated data for Zinc Oxide (ZnO) nanoparticles is evident through the PXRD pattern and crystallographic parameters (Fig. 2). The findings are supported by the reference code 01-076-0704 and the ICSD collection code 034477. The calculated peak list closely corresponds to the peak positions and relative intensities of the experimental data, indicating a high degree of concordance. This close correspondence strongly suggests that the crystal structure of the synthesized ZnO nanoparticles is in accordance with the anticipated hexagonal crystal system and space group (P63mc, space group number 186). Furthermore, the lattice parameters, specifically a = 3.2530 Å, b = 3.2530 Å, and c = 5.2130 Å, along with a calculated density of 5.66 g/cm^3^ and a unit cell volume of 47.77 × 10^6^ pm^3^, are the same for the prepared nanoparticles. The presence of two formula units per unit cell also anticipated from the reference structure, as indicated by a Z value of 2.00. Based on these compelling findings, it can be conclusively inferred that the ZnO nanoparticles were successfully prepared, and their crystal structure and characteristics are found to align with the provided information. The successful alignment between the experimental and calculated data provides confidence in the accuracy of the synthesis process and the resulting ZnO nanoparticles. This alignment also suggests that the synthesis method employed was effective in producing nanoparticles with the desired crystal structure and properties. The anticipated hexagonal crystal system and space group are known to contribute to the unique properties of ZnO NPs, such as their optical, electrical, and catalytic characteristics. In summary, compelling evidence is provided by the PXRD analysis of the ZnO NPs that the synthesis process was successful in producing NPs with the anticipated crystal structure and characteristics. The close correspondence between the experimental and calculated data, along with the agreement in lattice parameters, density, and volume of the unit cell, strongly supports this conclusion. These findings contribute to our understanding of the synthesis process and provide valuable insights for further research and applications involving ZnO nanoparticles.

The absence of AgO peaks in the XRD analysis can be attributed to several factors. It should be noted that the crystal structure of materials can be determined based on their diffraction patterns using the powerful technique of X-ray diffraction (XRD). However, certain limitations need to be considered when interpreting XRD data. Firstly, the limited sensitivity of XRD restricts its ability to detect low concentrations of a specific phase or impurity. If the amount of AgO present in the synthesized sample falls below the detection limit of the XRD instrument, the corresponding peaks may not be visible in the diffraction pattern. This lack of visibility could be due to the low concentration of AgO or its existence in a highly dispersed or amorphous form, which does not generate distinct diffraction peaks. Secondly, the reliance of the XRD technique on the assumption of crystallinity and long-range order in the sample is crucial. If the AgO phase exists in an amorphous or nanocrystalline form, the diffraction peaks may become broadened or smeared out, making it challenging to differentiate them from the background noise. Consequently, the diffraction pattern may not exhibit well-defined peaks corresponding to AgO. Additionally, XRD analysis is typically conducted on bulk samples, which may not accurately represent the entire composition of the sample. It is plausible that the AgO phase is localized in specific regions or exists as small clusters within the sample, eluding complete capture in the XRD analysis. Inhomogeneities or variations in the distribution of AgO within the sample can result in the absence of corresponding peaks in the XRD pattern. In conclusion, the absence of AgO peaks in the XRD analysis can be ascribed to factors such as the low concentration or dispersed nature of AgO, the presence of AgO in an amorphous or nanocrystalline form, localized distribution of AgO within the sample, and limitations of the XRD instrument. It is imperative to consider these factors when interpreting XRD data and to complement the analysis with other characterization techniques to attain an inclusive understanding of the composition and structure of the sample.

### Fourier transforms infrared spectroscopy (FTIR)

**Fig. 3:**
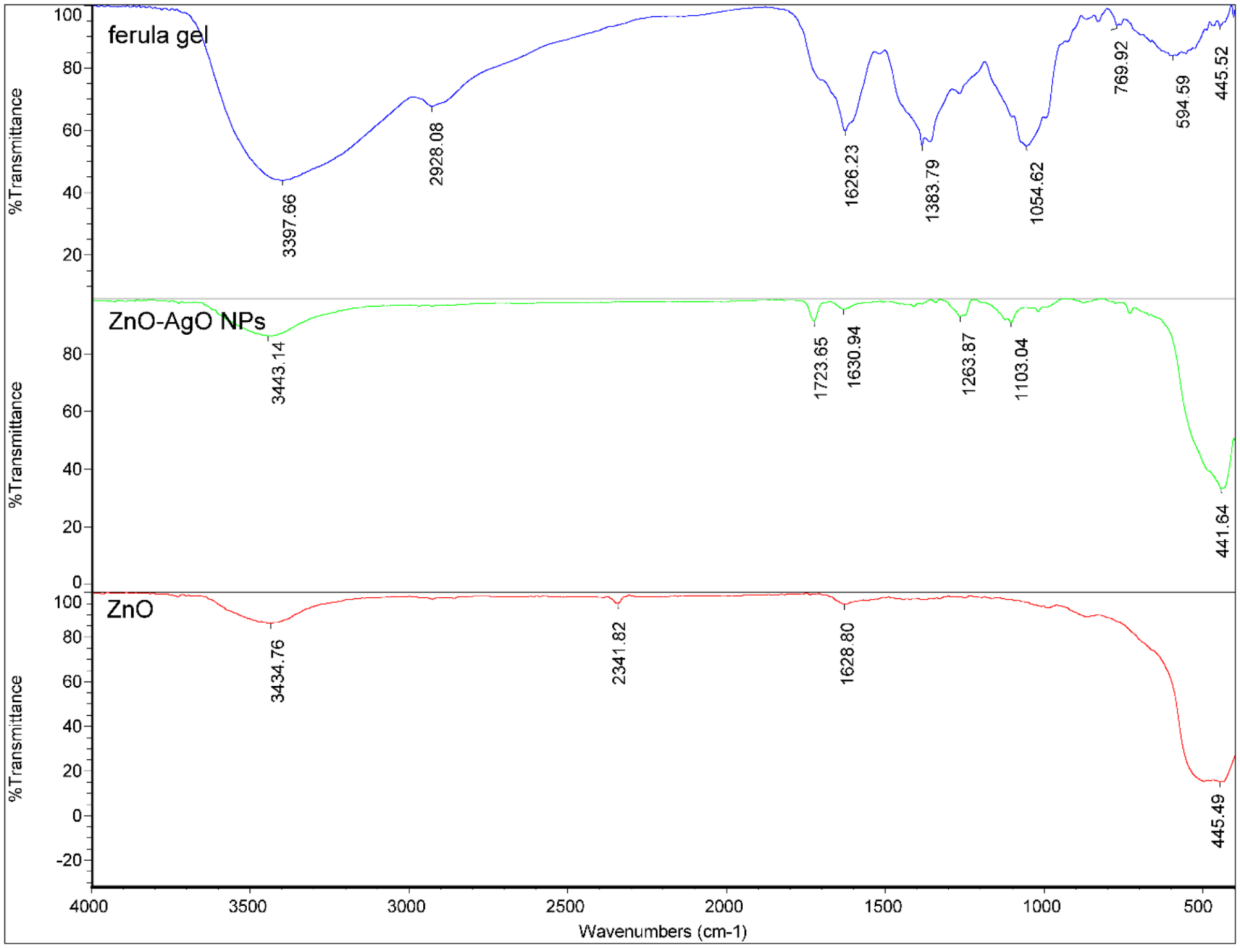
FTIR spectrum of *Ferula latisecta* gels, ZnO, and ZnO-AgO NPs

In this study, the FTIR spectrum of ZnO and ZnO-AgO NPs revealed a number of absorption bands (Fig. 3). The FTIR spectra of ZnO-AgO NPs and FTIR spectral analysis of the *F. latisecta* gel would be explained. In the FTIR spectrum of ZnO-AgO NPs, the band at 437.12 cm⁻¹ is likely associated with the stretching mode of Zn-O in ZnO, and the band at 726.38 cm⁻¹ could be linked to the bending mode of Zn-O-Zn in ZnO. The bands found at 872.94 cm⁻¹, 1019.91 cm⁻¹, and 1109.07 cm⁻¹ might be indicative of C-H out-of-plane and C-O stretching vibrations. The band at 1258.11 cm⁻¹ could be related to C-O stretching vibrations and –OH in-plane vibrations, while the band at 1412.69 cm⁻¹ might be due to O-H bending vibrations, –C–O–H in-plane bending vibrations, –CH_3_ out-of-plane bending vibrations, and –CH2– wagging and twisting vibrations. The bands at 1628.95 cm⁻¹ and 1725.04 cm⁻¹ could correspond to C = O stretching vibrations (possibly in the –COOR groups of crocetin or –COOH groups of amino acids), and the band at 3436.90 cm⁻¹ might be due to O-H stretching (possibly in alcohols or carboxylic acids). The FTIR spectrum also showed residual organic peaks, which could be explained by the ability of FTIR to detect changes in functional groups in biomolecules, thus identifying variations in the total composition of substances. This suggests that some organic materials from the Ferula latisecta extract may not have fully decomposed during heating, contributing to these residual organic peaks. The ZnO FTIR spectrum was also similar to ZnO-AgO NPs but showing lower organic residues.

The FTIR spectral analysis of the *F. latisecta* gel was characterized by several distinct peaks, each indicative of different functional groups. At 445.52 cm⁻¹, the peak was attributed to the vibrations of metal-oxygen bonds, suggesting the presence of metal oxygen compounds within the gel. The peak at 594.59 cm⁻¹ was associated with the bending vibrations of metal-oxygen bonds. Vibrations at 769.92 cm⁻¹ were indicative of C-H bending in aromatic compounds. The absorption at 1054.62 cm⁻¹ denoted C-O stretching vibrations, pointing to the presence of alcohols, ethers, or esters. A peak at 1383.79 cm⁻¹ suggested C-H bending vibrations in methyl groups. The strong absorption at 1626.23 cm⁻¹ was characteristic of C = C stretching in aromatic rings. At 2928.08 cm⁻¹, the peak indicated C-H stretching vibrations in methylene groups, while the broad peak at 3397.66 cm⁻¹ was typically associated with O-H stretching vibrations, likely due to water molecules, alcohols, or phenols. These functional groups collectively confirmed the gel’s potential as an effective capping agent for nanoparticle synthesis.

As for the absence of AgO peaks, it is hypothesized that this could be due to its low concentration or peak interferences. Factors such as unrelated chemical structural changes causing peak shifts and spectral interferences leading to difficulties in peak assignment could have contributed to the absence of AgO peaks in the observed XRD pattern.

### Field emission scanning electron microscopy (FESEM)

**Fig. 4:**
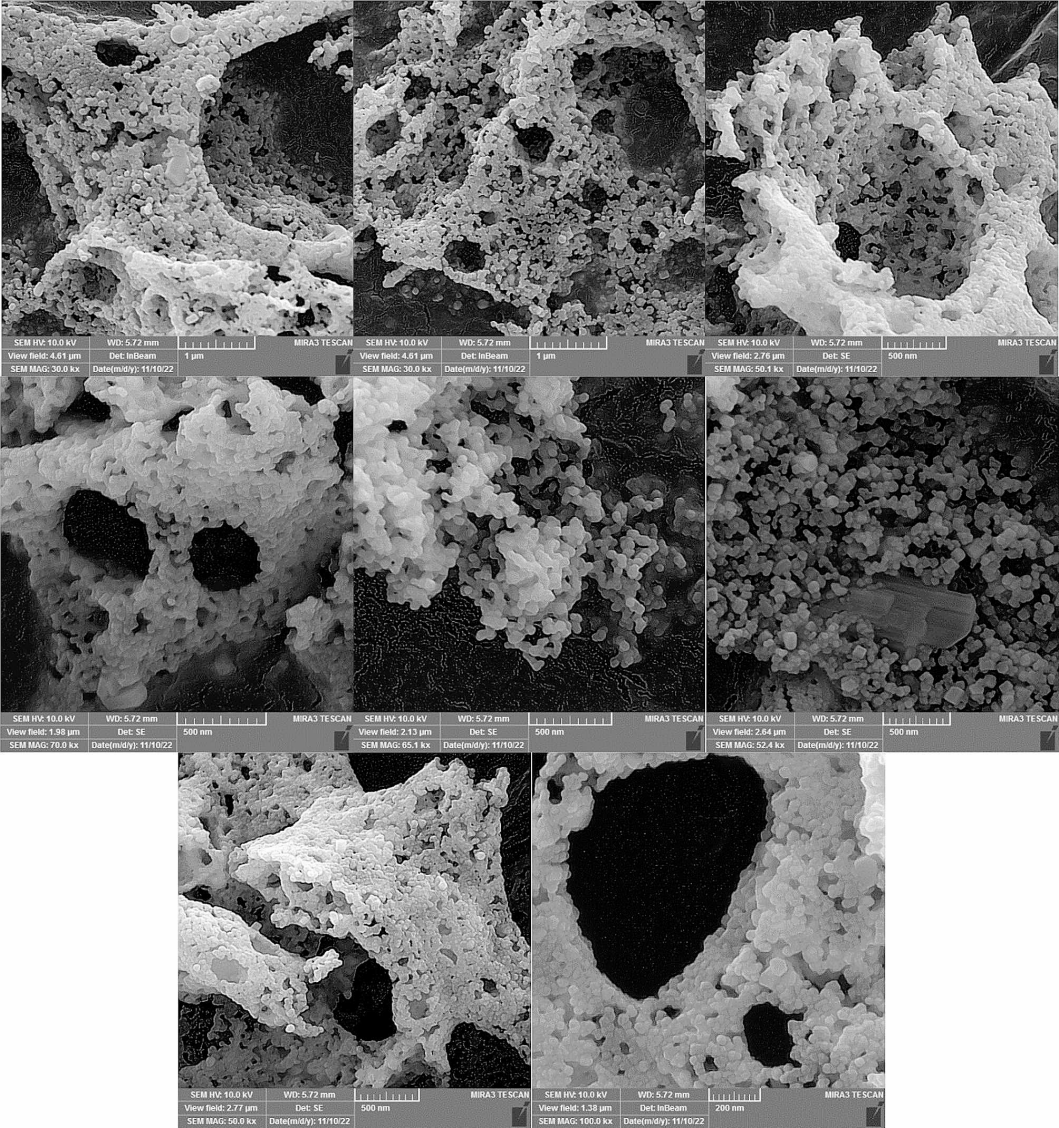
FESEM images of synthesized ZnO-AgO NPs

**Fig. 5:**
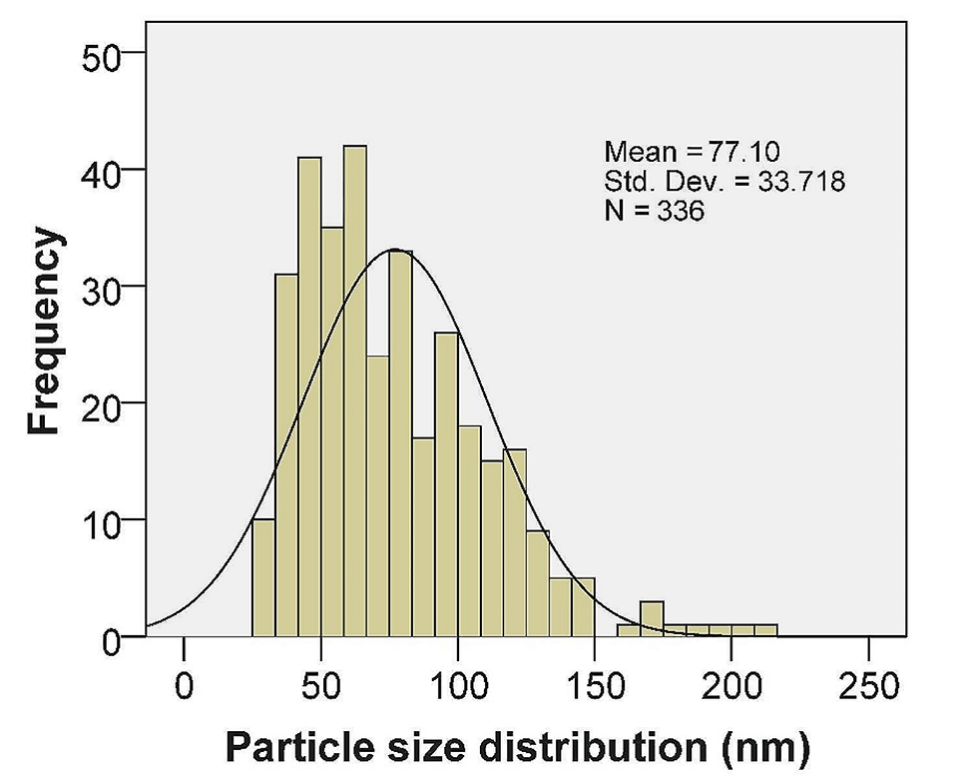
Particle size distribution of ZnO-AgO NPs

The results obtained from the analysis of the Field Emission Scanning Electron Microscopy (FESEM) image reveal important information about the particle size distribution and shape (Figs. 4 and 5). The particles observed in the image were found to be either spherical or semispherical in shape. The mean particle size was determined to be 77.1 nm, with a standard error of the mean at 1.8 nm. This indicates that the mean measurement is precise and provides a good estimate of the average particle size in the sample. To account for the presence of outliers, the median particle size was also calculated and found to be 69.5 nm. The median is a more robust measure of central tendency and is less affected by extreme values. Therefore, it provides a better representation of the typical particle size in the sample. The standard deviation, which measures the spread or dispersion of the particle sizes, was found to be 33.7 nm. This relatively high standard deviation suggests that the particle sizes in the sample are widely distributed, with a significant variation from the mean size. The range of particle sizes observed in the sample was 183.78 nm, with the smallest particle measuring 27.01 nm and the largest particle measuring 210.79 nm. This range indicates the diversity of particle sizes present in the sample. This suggests that the particles have a tendency to aggregate or agglomerate, cause the formation of larger constructions. Percentile analysis was conducted to further understand the distribution of particle sizes. For example, the 50th percentile (median) indicates that 50% of the particles have a size less than or equal to 69.5 nm. Similarly, the 75th percentile reveals that 75% of the particles are less than or equal to 98.1 nm in size. These results deliver appreciated understandings into the characteristics of the particles under study. The knowledge of particle size distribution and shape can be crucial for various applications and further research in fields such as materials science, nanotechnology, and particle engineering.

In this study, the quantification of nanoparticle (NP) size was typically performed using Image J, a versatile image processing software that allows for the analysis of various image parameters, including particle size. For statistical analysis, SPSS software is employed to interpret the data and provide insights into the size distribution and other relevant statistical parameters. In the SEM images, distinguishing between Ag and ZnO NPs based solely on visual inspection is challenging. However, when Ag is doped into ZnO structures, it may alter the morphology or create contrast in the images, which can be indicative of the presence of Ag. The encapsulation observed in the binary NPs could be due to organic residues that remained after calcination at 400 °C. While calcination typically decomposes organic matter, some compounds may resist complete breakdown and remain as a coating on the nanoparticles. Agglomeration in binary NPs can also occur for various reasons. Van der Waals forces, which are attractive forces between molecules, are a common cause of agglomeration. Additionally, during the drying process, capillary forces can pull particles together as the solvent evaporates. Electrostatic interactions and magnetic forces, if present, can also contribute to the agglomeration of nanoparticles.

### Energy dispersive X-ray spectroscopy (EDX)

**Fig. 6:**
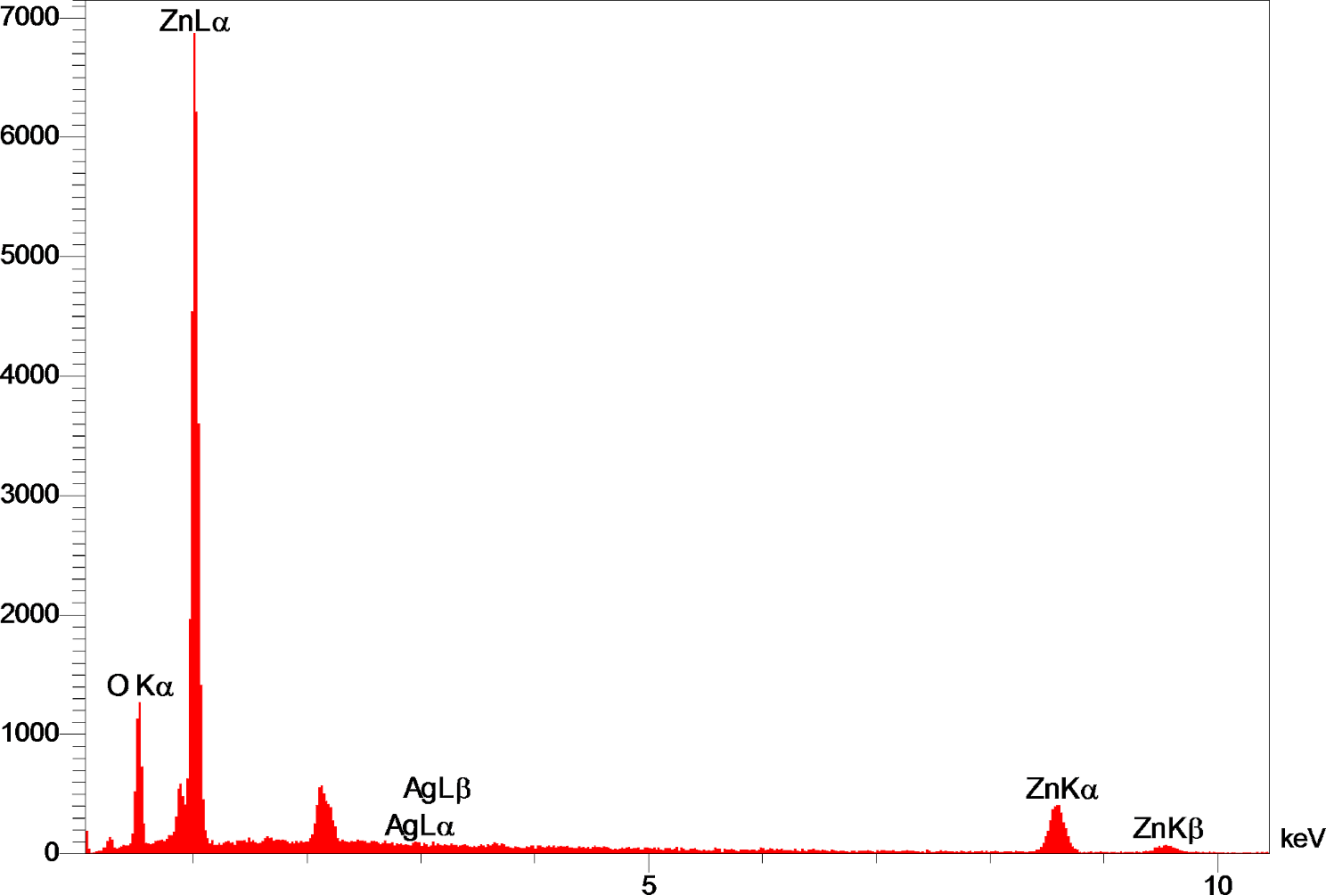
EDX analysis of ZnO-AgO NPs

In the analysis conducted by EDX, a variety of peaks were detected (Fig. 6), signifying specific electron transitions within the elements examined. Zinc (Zn) exhibited distinct peaks, namely Zn Lα, which corresponds to the electron transition from the L shell to the K shell, Zn Kα, representing the electron transition from the M shell to the K shell, and Zn Kβ, which is associated with the electron transition from the M shell to the K shell. Similarly, silver (Ag) displayed observable peaks, including Ag Lα, indicating the electron transition from the L shell to the K shell, and Ag Lβ, representing the electron transition from a higher energy level (M shell) to a lower energy level (K shell). Furthermore, oxygen (O) demonstrated a discernible peak denoted as O Kα, which signifies the electron transition from an outer shell (L or M shells) to the innermost shell (K shell). It is worth noting that no impurities were detected during the analysis. These peaks offer valorous insights into the electronic structure and behavior of the elements under investigation. The composition of the sample can be deduced from the quantitative data obtained from the EDX analysis. The sample is found to be rich in oxygen (O) and zinc (Zn), with oxygen being the predominant element (Table 2). The proportion of oxygen is roughly 23.93% by weight (W%) and 56.36% by atomic percentage (A%), whereas the proportion of zinc is about 75.11% (W%) and 43.30% (A%). A trace amount of silver (Ag) is also detected in the sample, contributing approximately 0.96% by weight (W%) and 0.33% by atomic percentage (A%). The findings from the EDX analysis suggest that the sample is primarily composed of zinc and oxygen atoms, leading to the inference that the chemical formula for the sample is ZnO, which represents zinc oxide. This indicates that the sample is predominantly made up of zinc oxide. As for the presence of silver (Ag) in the sample, it’s indeed possible to consider it as a dopant in the ZnO structure.

Doping is a widely used technique in semiconductor physics that involves the addition of impurities to a substance in order to modify its properties. In our specific study, we aimed to enhance the biomedical applications of ZnO by introducing silver (Ag) as a dopant. The incorporation of Ag into the ZnO structure has the potential to induce changes in the surface morphology and even deform the ZnO lattice structure. However, our experimental results, as confirmed by X-ray diffraction (XRD) analysis, did not exhibit any such effects. The XRD analysis revealed a ZnO structure with nearly prominent peaks, indicating the absence of impurities or other significant alterations. It is important to note that there are several possible scenarios that could have occurred but were not observed in our study. For instance, the introduction of Ag as a dopant could have resulted in a modified surface morphology or a distorted ZnO lattice structure. However, our experimental findings did not support these possibilities. Additionally, XRD investigation further confirmed that the presence of Ag had no discernible impact on the properties of the zinc oxide in ZnO-AgO NPs. Therefore, based on our research and the XRD analysis, it can be concluded that Ag can be considered a suitable dopant in this context, as it does not affect the properties of ZnO in ZnO-AgO NPs.

**Table 2:** Elemental composition of synthesized NPs

Elt	Line	Int	W%	A%	ZAF
**O**	Ka	96.4	23.93	56.36	0.4914
**Zn**	Ka	119.6	75.11	43.30	0.9212
**Ag**	La	5.1	0.96	0.33	0.7920
			100.00	100.00	

### Antimicrobial studies

**Fig. 7:**
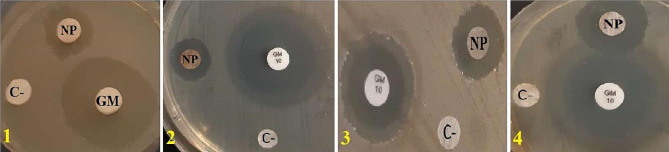
Disc diffusion Assay. The antibacterial activity of ZnO/AgO NPs towards infectional bacteria. The inhibition zones around the discs containing ZnO/AgO NPs showed their noticeable bactericidal activity against these pathogenic bacteria. NP: nanoparticle; C-(Plant materials as a negative control); GM (Gentamicin as positive control); **1**: E.coli; **2**: P.aeruginosa; **3**: B.subtilis; **4**: S.saprophyticus

According to the results of the disc diffusion assay, as shown in Fig. 7, the inhibition zones around the discs comprising ZnO/AgO nanoparticles showed the prominent bactericidal activity of these nanoparticles against the pathogenic bacteria assessed. The inhibition zones for *E. coli* and *P. aeruginosa* were 14.33 ± 0.1 and 13.66 ± 0.1 mm, respectively. For comparison, the inhibition zones for *B. subtilis* and *S. saprophyticus* were 15.66 ± 0.1 and 17.33 ± 0.1 mm, respectively.

In this experiment, gentamicin was employed as the positive control. It was notable that ZnO/AgO nanoparticles synthesized by *F. latisecta* displayed more potent anti-bactericidal activity against Gram-positive bacteria than Gram-negative bacteria. The inhibition zones detected in this investigation have been displayed in Table 3.

The MIC values for *E. coli* and *P. aeruginosa* (Gram-negative bacteria) were 100 and 125 µg/mL, and respective values for *B. subtilis* and *S. saprophyticus* (Gram-positive bacteria) were 100 and 75 µg/mL, respectively. The MIC values for *E. coli*, *P. aeruginosa*,* B. subtilis*, and *S. saprophyticus* were 125, 175, 125, and 100 µg/mL, respectively.

**Table 3:** Result of Disk diffusion, MIC and MBC analysis

Test	Material	*E. coli ATCC 25,922*	*P. aeruginosa ATCC 9027*	*B. subtilis PTCC 1365*	*S. saprophyticus ATCC 49,453*
**Disk diffusion**	ZnO/AgO NPs (300 µg/ml)	14.33 ± 0.1 mm	13.66 ± 0.1 mm	15.66 ± 0.1 mm	17.33 ± 0.1 mm
	Gentamicin (10 µg) [Control]	22 ± 33 mm	21.66 ± 0.1 mm	21.66 ± 00 mm	25.33 ± 00 mm
**MIC**	ZnO/AgO NPs	100 ± 00 µg/ml	125 ± 00 µg/ml	100 ± 00 µg/ml	75 ± 00 µg/ml
	Chloramphenicol [Control]	25 ± 00 ug/ml	25 ± 00 ug/ml	25 ± 00 ug/ml	25 ± 00 ug/ml
**MBC**	ZnO/AgO NPs	125 ± 00 µg/ml	175 ± 00 µg/ml	125 ± 00 µg/ml	100 ± 00 µg/ml
	Chloramphenicol [Control]	25 ± 00 ug/ml	25 ± 00 ug/ml	25 ± 00 ug/ml	25 ± 00 ug/ml

### Investigation of biofilm formation

Biofilm phenotypic investigation by titration microplates displayed that all established bacterial strains were able to produce a strong biofilm (Table 4).

**Table 4:** Optical absorption (OD_492nm_) of bacterial strains generating biofilm after 48 h

Bacterial strains and control	*E. coli ATCC 25,922*	*P. aeruginosa ATCC 9027*	*B. subtilis PTCC 1365*	*S. saprophyticus ATCC 49,453*	Control
**mean ± standard deviation**	2.006 ± 0.0007	2.402 ± 0.0014	1.712 ± 0.0019	1.801 ± 0.0004	0.303

### Minimal bacterial biofilm formation inhibitory concentration of ZnO/Ag nanoparticles

**Fig. 8:**
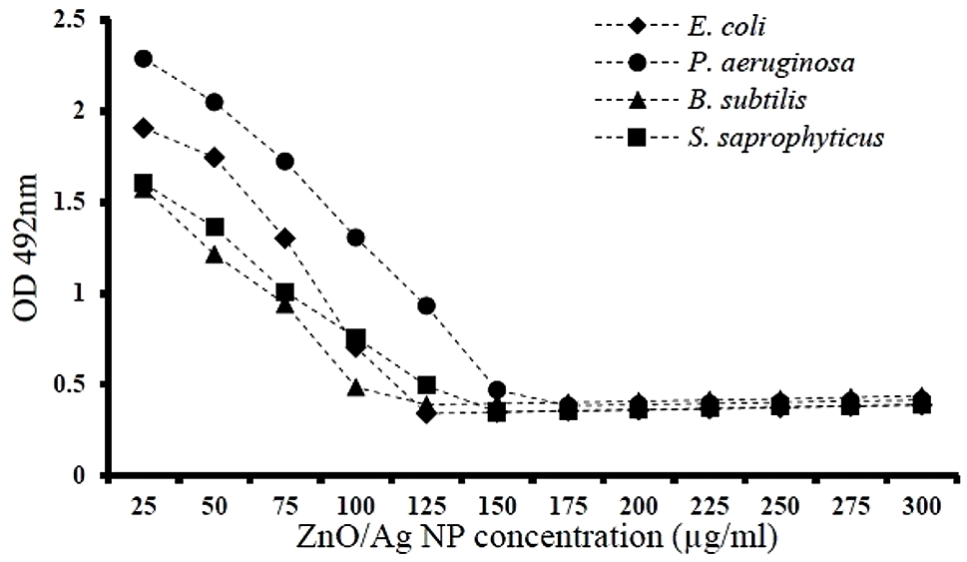
The effect of ZnO.AgO NPs on the inhibition of biofilm formation

**Fig. 9:**
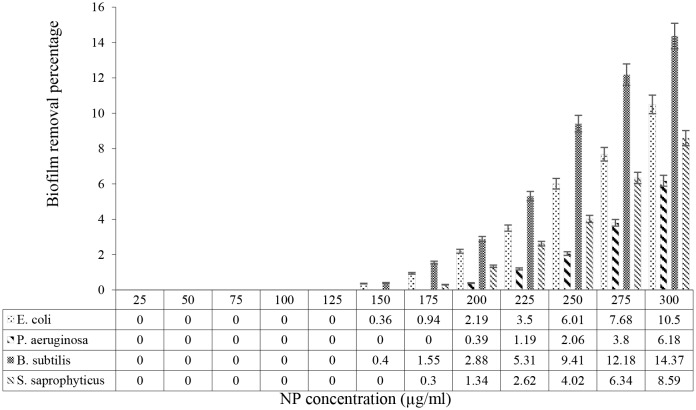
Removal percentage of biofilm formed in the presence of diverse dose of ZnO.AgO NPs

Biofilm formation was examined in the presence of diverse dose of ZnO/AgO nanoparticles by calculating average absorption at 492 nm (*n* = 3) to determine the capability of the NPs to inhibit biofilm formation. The consequences displayed that 492 nm absorption reduced by enhancing the dose of the nanoparticles, indicating that the NPs were able to prevent biofilm formation in a dose-dependent way. As can be seen in Fig. 8, the formation of biofilm was directly correlated with the dose of ZnO/AgO nanoparticles, and with an enhance in the dose of NPs, absorption at 492 nm decreased, reflecting a decrease in the formation of biofilm. As shown, after the complete inhibition of biofilm formation, from certain doses onward, the absorption started to increase compared to the control, which was insignificant and attributable to the presence of nanoparticles. According to results ZnO/AgO nanoparticles exert comparable anti-biofilm effects on Gram-negative and Gram-positive bacterial strains. According to Fig. 9, Zno/AgO nanoparticles were further able to eliminate the biofilm already formed by Gram-negative and Gram-positive bacteria in a dose-dependent way. As shown in Fig. 8, from the minimum biofilm inhibitory concentration toward lower concentrations, 492 nm absorption slightly enhanced compared to the control, which was caused by the presence of nanoparticles. Also, at the same concentration, nanoparticles more effectively eliminated the biofilm formed by *E. coli* vs. the biofilm formed by *P. aeruginosa*, and the same pattern was observed comparing the biofilm formed by *B. subtilis* vs. the biofilm formed by *S. saprophy*ticus. In general, the nanoparticles showed almost the same efficacy in eliminating the biofilm formed by Gram-negative compared to Gram-positive bacteria.

## Discussion

This study successfully synthesized zinc oxide nanoparticles (ZnO NPs) enhanced with silver (Ag) using *Ferula latisecta* gels as a stabilizing agent. The innovative use of plant gels not only offers a sustainable and eco-friendly approach but also imparts unique properties to the nanoparticles. The incorporation of Ag into ZnO NPs was found to significantly enhance their antimicrobial properties, as evidenced by the comprehensive antibacterial assays conducted against both gram-positive and gram-negative bacteria. The ZnO-AgO nanoparticles demonstrated remarkable antibacterial efficacy, with inhibition zones of 14.33 mm for *E. coli* and 15.66 mm for *B. subtilis* at a concentration of 300 µg/ml. The MIC values were determined to be 100 µg/ml for *E. coli* and 75 µg/ml for *S. saprophyticus*. These results indicate that the ZnO-AgO NPs possess potent bactericidal activity, particularly against gram-positive bacteria. The high surface area-to-volume ratio and small average size of the nanoparticles (77.1 nm) facilitate efficient interaction with bacterial cells, enhancing their antimicrobial activity. The enhanced antibacterial activity of ZnO-AgO NPs can be attributed to the synergistic effects of ZnO and Ag. ZnO NPs induce reactive oxygen species (ROS) production, leading to oxidative stress and cell membrane damage in bacteria. The presence of Ag ions further amplifies ROS production, resulting in heightened oxidative stress and bacterial cell death [70]. This synergistic mechanism makes it difficult for bacteria to develop resistance, addressing a critical challenge in antimicrobial therapy. Several researchers have suggested that biofilm is mainly disrupted as a consequence of Ag-ZnO NPs attaching to the extracellular polymeric substances (EPS) matrix of bacteria [71–73]. Different interactions between the metal oxides and the bacterial membrane cause physical disturbance, ions leakage, and the production of ROS, thereby producing oxidative stress and injuring chromosomal material [74–76]. The administration of Ag-NPs leads to changes in the morphology in the biofilm construction, such as the creation of cell surface abnormalities, cell wall disorders, penetrability differences within the membrane [77, 78].

This study highlights the excellent biocompatibility of ZnO NPs, making them suitable for various biomedical applications. However, it is crucial to thoroughly investigate the potential cytotoxicity of ZnO-AgO NPs on human cells. While incorporating of Ag enhances antimicrobial properties, it also raises concerns about toxicity at higher concentrations [79, 80]. Comprehensive cytotoxicity assays and in vivo studies are necessary to ensure the safe application of these nanoparticles in medical contexts. Using *F latisecta* gels for nanoparticle synthesis also offers several advantages. Plant gels contain bioactive compounds such as polyphenols, flavonoids, and terpenoids, which act as stabilizing agents. These compounds enhance the biocompatibility and therapeutic properties of the nanoparticles. The plant gel-mediated synthesis method aligns with green chemistry principles, reducing the need for hazardous chemicals and providing a sustainable approach to nanoparticle production.

Therefore, the adding of silver (Ag) into ZnO NPs has emerged as a game-changer, enhancing the antimicrobial prowess. Silver ions, known for their antimicrobial activities, synergize with ZnO NPs to significantly increase cytotoxicity against bacteria cells. The presence of silver ions elevates the production of ROS within cancer cells, leading to heightened oxidative stress and, consequently, bacterial cell mortality. This promising amalgamation elevates the effectiveness of ZnO NPs in antimicrobial treatment. Moreover, plant extracts, serving as green and eco-friendly agents in the synthesis procedure of ZnO NPs enhanced with silver (Ag), introduce a new dimension to the bactericidal properties of these nanomaterials. In biological production, plants reduce metal ions, and many biological factors present in the plant are effective in this process [81–83]. In this regard, the small size of nanoparticles has been introduced as an effective factor in antimicrobial action in various studies that have been conducted in the synthesis of different nanoparticles by plants [69, 84, 85]. Comparison of the present study with other studies shows that plant gel-mediated synthesized nanoparticles is in line with other studies and this method can be used as effective way for the synthesis of ZnO.AgO nanoparticles without the employ of chemical regeneration agents.

In recent years, drug resistance has been a global concern because the inappropriate and irrational use of antimicrobial drugs creates suitable conditions for the development of drug-resistant microbes. Infections caused by drug-resistant microorganisms do not respond to conventional treatments, and this leads to prolonged illness and increased risk of death. Nanomaterials are important due to their special characteristics such as high reactive activity and large surface/volume ratio. Nanoparticles are active under unfavorable conditions such as high sterilization temperatures, where conventional antibiotics are inactivated. On the other hand, it has been proven that metal oxide nanoparticles such as zinc have less toxicity in the body and due to their effective antibacterial activity against Gram-positive and Gram-negative bacteria, as well as the low cost of these nanoparticles, they can be a suitable candidate for combating and preventing growth of pathogens.

## Limitations

The limitations of this study were articulated with a focus on several key areas. The scope of antibacterial testing was limited, as only four bacterial strains were examined. These included *B. subtilis*, *S. saprophyticus*, *E. coli*, and *P. aeruginosa*, representing both gram-positive and gram-negative bacteria. A broader range of bacterial species, particularly clinically relevant pathogens and multidrug-resistant strains, should be considered in future studies to evaluate the antibacterial efficacy of ZnO-AgO nanoparticles. Mechanistic insights provided by the study were incomplete. Although the enhanced antibacterial properties of ZnO-AgO nanoparticles were demonstrated, the interactions with bacterial cells at the molecular level were not fully elucidated. Detailed mechanistic studies, potentially involving advanced techniques such as proteomics, transcriptomics, and metabolomics, could offer deeper insights into the pathways through which reactive oxygen species (ROS) are generated and lead to bacterial cell death. Potential toxicity concerns were briefly mentioned but not thoroughly investigated. The biocompatibility of ZnO nanoparticles was noted, yet the potential cytotoxicity of ZnO-AgO nanoparticles on human cells was not extensively studied. Comprehensive cytotoxicity assays, including long-term exposure studies on various human cell lines, are essential, along with in vivo studies to assess potential toxicity and biocompatibility in animal models. Variability introduced using plant materials, such as those from *F. latisecta*, was acknowledged. Factors like the plant’s geographical origin, harvest time, and extraction method can affect the concentration of bioactive compounds, leading to inconsistencies in nanoparticle synthesis and properties. Standardizing the extraction process and characterizing the plant extracts for their bioactive components are crucial to minimize this variability. The stability and shelf-life of the synthesized ZnO-AgO nanoparticles were not addressed. Nanoparticles alteration situations, such as aggregation, dissolution, or alterations in their physicochemical properties, could be impact their antibacterial efficacy. Systematic studies on the stability of these nanoparticles under various storage conditions and over extended periods are necessary. The lack of in vivo efficacy data was also noted. While in vitro antibacterial activity was demonstrated, the complex in vivo environment and factors like the immune response, nanoparticle distribution, and potential side effects are critical in determining therapeutic efficacy. Animal studies are needed to evaluate the in vivo antibacterial activity and safety profile of these nanoparticles before clinical trials can be considered. Challenges related to scale-up and manufacturing were also important. The synthesis process described on a laboratory scale may present significant challenges when scaled up for industrial or clinical use, including issues with batch-to-batch consistency, cost-effectiveness, and regulatory compliance. Developing scalable and reproducible manufacturing processes is essential for commercial viability. Regulatory and ethical considerations were not discussed in the study. Nanoparticles in medical applications must adhere to stringent regulatory standards to ensure patient safety. The regulatory pathway for ZnO-AgO nanoparticles, including necessary preclinical and clinical evaluations, as well as the environmental impact of nanoparticle production and disposal, should be considered. Lastly, the potential interactions between ZnO-AgO nanoparticles and other medications were not explored. In clinical settings, patients often take multiple medications, and understanding how nanoparticles interact with other drugs is critical to avoid adverse effects and ensure therapeutic efficacy. Studies investigating these interactions are necessary for the safe integration of nanoparticles into medical treatments. In conclusion, while the study presents promising advancements in the synthesis and antibacterial application of ZnO-AgO nanoparticles using *F. latisecta* gels, addressing these limitations is crucial for their successful translation from the laboratory to clinical and industrial applications. Future research should focus on expanding the spectrum of tested bacteria, providing detailed mechanistic insights, ensuring comprehensive safety evaluations, standardizing plant extract usage, and addressing scalability and regulatory challenges. By overcoming these limitations, the potential of ZnO-AgO nanoparticles in combating bacterial infections and drug resistance can be well known.

## Conclusion

In conclusion, the study demonstrates the successful synthesis of ZnO-AgO nanoparticles using *Ferula latisecta* gels, highlighting their potent antibacterial properties and biocompatibility. The innovative synthesis method aligns with sustainable practices, offering a promising approach to addressing drug-resistant bacterial infections. However, addressing the identified limitations through comprehensive research is essential to understand the potential of ZnO-AgO NPs in biomedical applications. Future studies should focus on expanding the antibacterial spectrum, understanding the mechanisms of action, ensuring safety, and developing scalable manufacturing processes. By overcoming these challenges, ZnO-AgO nanoparticles could emerge as a powerful tool in the fight against bacterial infections and drug resistance.

## Data Availability

No datasets were generated or analysed during the current study.
